# The acceptability, practicality, implementation and efficacy of a physical and social activity intervention ‘BreatheHappy’ for people with long-term respiratory conditions: A feasibility study

**DOI:** 10.1177/14799731241238435

**Published:** 2024-03-29

**Authors:** A Lewis, LA Turner, S Fryer, R Smith, H Dillarstone, YW Patrick, E Bevan-Smith

**Affiliations:** 1 Department of Health Sciences, 3890 Brunel University London, Uxbridge, UK; 2 School of Health Sciences, University of Southampton, Southampton, UK; 3 School of Education and Science, 2376 University of Gloucestershire, Cheltenham, UK; 4 Department of Geography, 4919 University College London, London, UK; 5 Institute for Global Health, 4919 University College London, London, UK; 6 Department of Health and Social Care, 2376 University of Gloucestershire, Cheltenham, UK

**Keywords:** Pulmonary rehabilitation, physical activity, sedentary behaviour, qualitative

## Abstract

**Objectives:**

This study aimed to determine the feasibility of a group-based pilot programme of low-to-moderate physical activity training, education and social activities, by investigating acceptability, practicality, implementation and efficacy testing. We offer suggestions on programme adaptions for future study.

**Methods:**

People with a range of chronic respiratory diseases were invited to participate in a pilot 12 week group activity programme. Activities included outdoor walking, tai-chi, education and a range of social activities. Acceptability was determined by participant experiences determined during interviews. Practicality was determined by programme and outcome measure completion, cost and adverse events. Implementation was determined according to whether the programme ran as planned. Efficacy was determined by statistical analyses of outcomes including hand grip strength, timed up and go test, COPD Helplessness Index, COPD Assessment Test, and measures of physical activity via accelerometry.

**Results:**

Thematic analysis indicated that the “BreatheHappy” programme was acceptable. Seven of nine participants completed eight out of 10 sessions and the majority completed all outcome measures. “BreatheHappy” was therefore considered practical. The programme was not implemented as planned, with only 10 sessions running rather than the 12 intended. There was a significant increase in daily step counts (MD: 1284 95% CI: 240-2329 *p*: 0.024 effect size: 0.988), stepping time (MD: 16 min 95% CI: 5-27 min *p*: 0.011 effect size: 1.36) and daily minutes completing light physical activity (MD: 23 95% CI: 6-38 *p*: 0.006 effect size: 1.6). However, time spent sitting for ≥30 min but ≤60 min significantly increased (MD: 26 95% CI: 0.2-52 min *p*: 0.049 effect size: 0.931), showing signs of efficacy and changing physical activity behaviour patterns.

**Discussion:**

A 10-week programme of low-moderate physical activity training, education and social activities shows signs of feasibility for future research. Suggested adaptions for future study include using physical activity measures such as daily step count or light physical activity for a primary outcome, and mental health and social health related outcome measures relatable to participant's beneficial experiences of the programme. Recruitment in future studies will try and reach both those less socially active and possibly those who have completed pulmonary rehabilitation (PR). Venues should be close to efficient transport links whilst different frequencies and durations of programme delivery should be trialled. Adequate funding should be provided for both staff running the programme and blinded research staff for outcome measurement.

## Introduction

Chronic lung diseases, such as Chronic Obstructive Pulmonary Disease (COPD), are a huge burden to health and social care services costing thousands of pounds per patient per year, and for the UK this equates to over 46 billion pounds over 20 years.^1–3^ The economic cost of Asthma is approximately six billion pounds a year in the UK .^4^ According to economic analyses, Pulmonary rehabilitation (PR) is a hugely valuable intervention regarding cost per quality-adjusted life years for people with chronic respiratory disease.^5,6^ PR is a cornerstone gold-standard intervention shown to reduce mortality, improve exercise capacity, breathlessness and quality of life.^7,8^ One of the aims of PR is to improve patient physical activity, with limited data showing effectiveness and the components and models of interventions to improve physical activity remaining unclear.^9–11^

PR programmes are not always easily accessible and there are high reported patient non-completion rates.^12,13^ Factors associated with poor uptake and non-completion of PR are multiple and diverse, such as being more breathless, having a higher social deprivation status, transport issues and health system resources, referral processes, and patient beliefs about the lack of perceived benefit and the value of other activities that would be missed if attending PR.^14–16^ There is a need to address this multi-faceted problem. An American Thoracic Society/European Respiratory Society working party in 2015 recommended development of PR models that were more accessible and acceptable for patients.^17^ Furthermore, the NHS Long Term Plan indicates that alternative PR models need to be tested and implemented in the UK, including those that support self-management.^18^ Importantly, providing a more personalised, accessible and flexible model of rehabilitation may address long-term adherence to behaviours that enhance physiological, psychological and social health-related outcomes, and increase patient access to interventions for a diverse population.^19,20^ The “BreatheHappy” model of rehabilitation offers a community-based programme using minimal equipment and resource, targeting potential barriers of PR, to increase access and completion of a group-based programme, and increase physical activity with transferable maintenance into home settings with patient independence.

“BreatheHappy” began as an informal local weekly incremental walking, physical activity and social support group. Informal observation and participant feedback indicated that the intervention is effective in increasing activity, ‘happiness’, self-esteem, building confidence and reducing the perception of vulnerability, significantly improving the life of participants. Unlike PR, which has a structured, prescribed, moderate-to-high intensity exercise programme at its core, “BreatheHappy” has low to moderate physical activity as one of its cornerstones. It is socially focussed and addresses intensely the psycho-social dysfunction experienced by participants. Indeed, while high-intensity, high-frequency (2-3 sessions/week) supervised exercise sessions are efficacious in eliciting physiological changes in patients with respiratory disease,^21^ low-intensity, lower-frequency (1 session/week) supervised activity may promote, longer-term activity adherence and associated health-related outcomes.^22^ Tai-Chi Movements for Wellbeing (TMW) is a newly developed intervention that has been modestly demonstrated to be helpful and enjoyable for people with COPD.^23^ TMW offers a reduced sequence of movements which are easier to learn and people are able to participate in with physical disabilities, that still focus on ‘grounding’ through the legs and feet (thought beneficial for lower limb endurance) and rhythmical breathing with movement (thought beneficial for dyspnoea). In “BreatheHappy”, TMW supports the physical activity element by attention to grounding, core stability, confidence and the ‘soft limits’ of ability.

Prior to undertaking a large-scale clinical effectiveness trial, it is necessary to define the feasibility of the programme according to the following objectives aligned to previous recommended criteria.^24^

### Acceptability

Our objective was to consider the extent to which the programme was deemed acceptable from people who participated in the programme. Acceptability was put in context with living with a respiratory condition and people’s daily life activities.

### Practicality

Our objective was to determine the extent to which the programme can be completed by participants and how practical and burdensome it is to collect research outcome data with the available resources and funding.

### Implementation

Our objective was to determine the degree to which the “BreatheHappy” programme was implemented as proposed, and understand reasons for when the programme was not implemented as intended. A further objective here was to collect data to help make changes in consideration of future trial design, should funding be awarded.

### Limited efficacy testing

Our objective was to investigate signals of efficacy from a novel programme participation with a novel outcome measure set. The objective was to refine the choice of a primary outcome measure, (should any show efficacy), which may be used for sample size estimation for a future study, using effect size estimations from this study.

### Adaption

Our objective was to test the use of a range of outcome measures to contemplate necessary adaption of outcome measures of value for a future study. Another objective was to provide suggestions for programme adaptions based on the meaning of participant experiences, to optimise the design of a future clinical effectiveness trial.

## Methods

### Design

The study was a prospective mixed-methods cohort design.

#### Acceptability

Acceptability was determined via qualitative interviews with participants living with lung disease. Qualitative data, related to participants experiences of “BreatheHappy”, were collected using face-to-face, online and telephone interviews conducted by two researchers who were unknown to the participants and trained in interview techniques. Interviews comprised of semi-structured questions (See Appendices) and were digitally recorded. The researchers took notes during interviews and transcribed verbatim. Reflexive thematic analysis was performed, which was predominantly inductive.^25^ The choice to use reflexive thematic analysis over other thematic analysis choices provided the opportunity for researchers to challenge their own understandings and pre-conceptions within the data analysis and use their own experiences of participating in different activities in the interpretation of participant narratives. There were two researchers, one researcher performed online interviews (HD), and the other researcher (RS) performed the interviews face-to-face as it was thought important to offer participants a choice of interview method. Because two researchers performed the interviews both were involved in the analysis for reflexive value in the analysis. Group discussions were then had with staff with qualitative research experience (AL) to challenge and develop interpretations and analysis before final themes were decided. Member checking was not necessary as the contextualist and reflexive nature of this work valued researcher interpretation within the analysis. Furthermore, the concept of data saturation was not considered relevant as this was not based on grounded theory methodology and we did not perform theoretical sampling.

#### Practicality and Implementation

Practicality was determined primarily by participant completion of the programme. Completion of the programme was determined at a 75% completion of sessions (8 out of 10 group activity sessions). Practicality was further determined by the completion of paired outcome measures which were patient reported and objectively determined performance-based measurements. Finally, implementation was determined by whether all sessions ran as planned. The programme was intentionally flexible so that this could be more person-centred, adaptable and responsive to the participants joining the group. One of the objectives was to determine which activities were more valuable to patients rather than have a pre-determined programme. Therefore, the sessions in the protocol were a framework rather than set in stone. Consequently, further traditional measures of fidelity were not deemed appropriate.

A convenience sample of participants with a diagnosis of chronic respiratory disease were recruited via a number of routes including the local Asthma and Lung UK Breathe Easy group and local members of the public responding to a social media advert. Inclusion and exclusion criteria are reported in Table 1. Participants were able to invite a maximum of one carer to attend “BreatheHappy” and participate in the interventions on a supportive basis only.

**Table 1.** Inclusion/exclusion criteria for “BreatheHappy” study.

Parameters	Inclusion criteria	Exclusion criteria
Diagnosis	• Self-reported diagnosis of a chronic respiratory disease to include COPD, Bronchiectasis, asthma, interstitial lung disease, and lung cancer	• An existing co-morbidity deemed to introduce a high level of risk when taking part in low grade physical activities. These may include, for example, unstable angina or severe dementia.
Commitment	• Willing to commit to attend weekly group sessions regularly and assessment appointments.	• Not able to commit to attending regular group sessions.
Ability	• Able to physically attend the venue • mMRC 2 and above	
Pulmonary rehabilitation		• Due to start pulmonary rehabilitation within 12 weeks of start of study • Eligible for pulmonary rehabilitation and have access to a local programme but have never been offered a referral.
Blood gas status	• Participants able to maintain target oxygen saturations with or without ambulatory oxygen.	• Oxygen saturations consistently fall below target saturations at rest or on walking who do not have and oxygen assessment appointment within 3 weeks of programme commencement.

Hand Grip Strength and Timed Up and Go Test data were collected at the start (Baseline) and end of the intervention during week 1 and 10, respectively. The physical activity data and sedentary behaviour data were collected between weeks one and two (before activity intervention started) and between weeks 9 and 10 (after activity intervention ended) (see Online Appendix Figure 1 of the schematic timeline of the programme). Psychological status and quality of life was assessed using the COPD helplessness Index (CHI)^26^ and COPD assessment test (CAT) score with an established MCID of two in relation to PR.^27^ Quantitative function assessments were completed using the Timed Up and Go (TUG) test with MCID of 1.4 in response to PR^28,29^ and handgrip strength (dominant and non-dominant hand)), previously shown to show a significant positive relationship with quality of life for individuals living with COPD,^30^ with an estimated MCID of 5 kg.^31,32^ Standardised instructions for the participants and protocol for staff were followed and are provided in the Appendices.

Free-living physical activity was assessed using GENEActive (Activinsights Ltd, Newcastle, UK) accelerometers. The GENEActive, is a wrist-worn (non-dominant), triaxial accelerometer that provides valid and reliable measurements of physical activity intensity classifications.^33^

The GENEActive was configured to record at a sampling frequency of 50 Hz, with files extracted using the GENEActivee software (version 3.3). Generated. bin files were processed and analysed through R using GENEActive R markdowns (Activinsights Ltd, Newcastle, UK). Activity reports for total time spent in sedentary (<1.5 METs), light (1.5 – 3.99 METs), moderate (4.0 – 6.99 METs) and vigorous (>7 METs) intensity activity were reported using previously validated cut-offs.^33^

Total sedentary time, patterns of sedentary behaviour and postural transitions were assessed using the activPAL 4 (PAL Technologies, Glasgow, Scotland). The activPAL monitor is a triaxial accelerometer that demonstrates valid and reliable measures of posture and motion in everyday living, including sitting/lying, standing, step count, sit-to-stand transitions and walking.^34^ The monitor was placed in a waterproof, nitrile sleeve before securing to the participants right thigh (mid-anterior) using a waterproof dressing (Tegaderm^TM^). Data was recorded at a sampling frequency of 10 Hz for each 24 h period (midnight to midnight) and then downloaded and processed using activPAL software (PAL analysis version 8.11.8.75).

Participants wore both the GENEActive and activPAL monitors, continuously for 6 days between sessions 1-2 (baseline) and sessions 9-10 (end-intervention). Participants recorded sleep and non-wear time in a diary to detect invalid days in combination with monitor data, invalid days (waking wear time <10 h) and any data recorded during the intervention sessions were subsequently removed from analysis. Average values across the valid days are reported for all dependent physical activity and sedentary behaviour outcome variables.

The activPAL accelerometer has been validated on low-intensity activities (sitting, lying, sit-to stand) and sedentary time in free-living conditions in older adults. While the activPAL has been used in the measurement of physical activity, there is evidence to suggest that the activPAL may overestimate energy expenditure as the algorithm is based on step count. This is particularly true for high-intensity physical activity. Literature suggests therefore that to quantify sedentary behaviour and activity levels in older adults in a community setting, a wrist-worn accelerometer should be incorporated alongside an activPAL.^35^

Literature suggests that 5-valid days of sedentary monitoring with the activPAL provide reliable estimates of these outcome measures.^36^ In the current population, data collection was focused on physical activity in the light-to-moderate intensity domain, therefore, Dillon et al ^37^ suggest 2-valid days of activity monitoring with the GENEactiv.

Practicality was further determined by programme uptake, costs and adverse events.

Project costs were defined in terms of staff costs which included face-to-face contact time and administration. These costs came out of funds directed from the Grant via the University teaching team and were equivalent to costing x 2 NHS AFC Band seven clinicians for the programme contact hours combined with the administration. The other project costs were defined under room hire and “Miscellaneous” which consisted of stationary and refreshments.

An adverse event was defined as any event where the patient experienced harm during their attendance period at “Breathehappy”. This may include physical harm from trips, falls or MSK injury from undertaking physical activity, or psychological harm, for example from a panic attack.

#### Limited efficacy testing

Statistical analysis of the quantitative data was performed using Statistical Package for Social Sciences Data version 28 (IBM, Chicago, IL). Following the Shapiro-Wilk test, all dependent variables except for time spent in vigorous and moderate physical activity, and sedentary bouts ≥2 h and ≤4 h, were found to be normally distributed. Paired samples *t*-tests were used to assess baseline versus end-intervention changes in psychological, functional, quality of life, physical activity and sedentary behaviour. Data are reported as mean, standard deviation (SD), mean difference (MD) and 95% confidence interval (CI). Where data was not normally distributed, a Wilcoxon signed rank test was used with data reported as median score and interquartile range (IQR). Cohens’ *d* is reported as a measure of effects size, where 0.15, 0.4, and 0.75 represented a small, medium, and large effect respectively, and Pearson’s R effect sizes of 0.10, 0.20 and 0.30 accordingly, α was set a priori at *p* < .05.^38^

#### Adaption

Necessary adaption of the “BreatheHappy” programme was considered once programme patient-reported and performance outcome data and qualitative data were made available so these could be triangulated and suggestions made, for possible future programme adaption regarding “BreatheHappy” programme structure, content and delivery. These suggested adaptions are made in the discussion section of this paper.

Quantitative data were collected at the “BreatheHappy” venue. The venue provides an indoor space on the periphery of a natural environment suitable for outdoor activities and parking was available adjacent to the venue.

### Intervention

The planned “BreatheHappy” programme was a structured group intervention lasting for 2 hours, once a week for 14 weeks (See programme protocol in appendix). The programme focussed on a menu of physical activity and social connectivity options comprising group walking, TMW, walking sports, group self-management discussion, education and problem-solving activities. Besides other social activities that participants were free to participate in, which may have impacted on the outcomes in this study, individuals were not current participants in a PR programme during their study participation. The Breathtec digital programme^39^ was a resource made available free of charge to all participants who were all signposted to the site and invited to use the resource in tandem with the programme.

Institutional ethical approval was granted (University of Gloucestershire’s Research Ethics Committee REC.22.53.1). All participants provided written informed consent.

## Results

### Acceptability

All nine of the participants agreed to participate in the qualitative interviews. Three interviews were performed face-to-face and six remotely. The average interview time was 48 min. Four themes related to broader experiences of living with a chronic respiratory disease including (1) Having coping mechanisms, (2) Frustration, (3) Knowing own boundaries, and (4) Lack of respiratory care in the NHS. Five themes related specifically to the experiences of “BreatheHappy”: (1) A valuable social life, (2) Disease education beneficial for self-help, (3) Improved mental health, (4) Supportive leadership, and (5) Logistics of “BreatheHappy”. Quotes associated with each theme are presented in Table 2.

**Table 2.** Quotes from participants.

Theme	Quotes
Having coping mechanisms	“Well, when it first happened, you know, when you have difficulty in breathing you tend to panic to start with, and of course that makes things worse. But now I’ve long accepted it and just carry on.” (P1) “I never go anywhere without my inhalers” (P6) “I’ve had different techniques and I got into this technique because I remember since I didn’t do any yoga as such but they were talking about keeping this all open so then I started literally putting my hands behind the back of my neck and watching the telly like that, you know what I mean, or it can be annoying but even if you’re at the cinema and somebody’s sat in front of you like me like this but I even do it today and I find it’s the only position that really works for me. So I go for a walk on the (name) Hills 3 or 4 times a week sometimes more, so even if I’m walking up, and if I can I’ll walk like this because I know that this-, because my lungs are so scarred, there’s very little tissue keeping them open…So what I’ve noticed is if I’m in this position on a walk like this and I’ll make sure nobody’s around” (P8)
Frustration	“I’m sick of it and I do think a lot of people can get, umm, I was depressed for quite a few years ago because I was just sick of it, I wasn’t suicidal, but I’d wake up dreaming of hanging myself, I was just sick of it” (P8) “I’ve always thought well it’s just asthma and that’s how I’ve been made to feel, I think, it’s only asthma” (P5) “I guess people not understanding is quite frustrating, um yeah, but emotionally like I said I’ve got nothing to compare it to really” (P5)
Knowing own boundaries	“I’d love to do it all but I know it’s beyond me, I know it’s beyond me….I know I couldn’t do it, I couldn’t jog to that white car, I’d be [breathless actions] could march there quite happily and I would want to as well.” (P8) “Well, when it first happened, you know when you have difficulty in breathing you tend to panic to start with, and of course that makes things worse. But now I’ve long accepted it and just carry on.” (P1)
Lack of respiratory care in the NHS	“there seems to be a gap for lungs [in the NHS], and yet breathing is essential” (P2) “I, I sometimes wonder why I didn’t find out about [BreatheHappy/BreatheEasy] from the surgery. You know, there could have been a notice in the surgery. I’ve never seen that in the three surgeries I’ve been in since it was diagnosed. So, is it the fact that the doctors don’t think it’s important? Or… I just don’t understand. That’s the easiest thing in the world to put up a notice in the surgery.” (P2) “because if you’re reliant on the doctors or nurses to tell you anything you wouldn’t, you wouldn’t be aware of anything, and I’m not the only person saying that, lots of people have said it” (P7)
Valued social side of BreatheHappy	“that’s what makes you do stuff but it’s, for people in the same boat as me, you know, I like holding that worse, but just exercise.” (P3) “it’s good here and it’s, it’s gone so quickly. It’s weeks. I can’t believe,… just nice being around with others. No. Here we go, but different complaints. But stopped. It’s just, I’m just sad. (Its) Finished”. (P3) “There’s other people there that you interact with… which I don’t get really at other times, you know, because I don’t really see anybody much” (P4) “it’s not like you ever meet other people with bad lungs and if you meet someone with bad lungs you don’t talk about it, it’s not like they support the same football team.” (P8) “Yeah, I haven’t really met many people with a condition like mine, I didn’t really know anyone with bronchiectasis, um, and people, a lot of people say they’ve got asthma but use their inhaler once a month or something, and I don’t think people realise how debilitating asthma can be when you’ve got it like I’ve got it so the group has been useful to meet people that have got a similar condition” (P6) “Well, you meet people there and um, it’s the one thing I like about it is the social part. There’s no one there that I knew as friends before, but it’s yeah, the social part I enjoy” (P1) “So yes, that’s been nice as well because it’s been done with other people at a similar pace to me. Whereas before I would probably have not joined in those things because I couldn’t have kept up with people if that makes sense.” (P5)
Disease education beneficial for self-help	“so I’ve always just like been seen by an asthma nurse or a GP if it’s gone to the point where I need steroids or antibiotics. But I’ve never had it explained to me what triggers it and and, um, how to do the breathing control to calm it down. And also know about different foods you can eat, and we went into many subjects. So yeah, it’s been very beneficial in that-, to have it explained what happens while-, why you have these attacks and yeah I’ve never had that before. And also talking about different medications and, yeah” (P5) “It’s much more informative. People more. And more of a group rather than a couple of speakers… well anything to help with the condition really, many many ideas. Very different. I think you many people have understand why. I’m just thinking to help yourself. Umm, That’s why I’m doing it. (P3) “You forget stuff, so you grow out of it and stop doing it, and then suddenly some more information comes along and you go ‘oh yeah I remember that, that’s right’, and that’s how it’s been really so it’s sort of sparked stuff” (P8)
Improved mental health	“I think my well-being is better when I’m there and when I’ve been, you know. But physically doesn’t make that much difference to me really” (P4) “I think it’s helped my mood consistently” (P5) “it’s made me more motivated and more determined to keep active” (P9)
Supportive leadership	“Yeah, I think the difference is it was run by (name) who we could interact with, ask questions, get advice, umm, everyone that came along to the talk was relevant to our condition” (P5) “I’m a big fan of (name), but I do think that it takes someone as special as (name) to make you want to go, and another person might not, another leader might not be the person to do it if you know what I mean.” (P9) “she explains everything so precisely and so understandably and you know, she, she talks in a, in a way that everybody can understand, and if you don’t understand, you just ask her and she’s, you’re not afraid to ask. She is so approachable and so easy to understand” (P9)
Logistics of BreatheHappy	“If it was location, I think the only thing I would change is, is location” (P8) “I wish it was longer” (P5) “just more, more, more, more weeks. Yeah. Week personally. OK. So more weeks in total” (P3) “And maybe for the future, it needn’t be once a week, it could be once a fortnight, once a month… Well, on an ongoing basis because umm, there are lots of things to do and you can’t do them all, so if it was every week, you’d be cutting out something else.” (P2) “Yeah. it was good because I could join in and I wouldn’t have been able to, but it’s not as good as being in person. But I think we’ve all got used to Zoom things anyway but um yeah, it was a bit more difficult doing the Tai Chi I thought, well if you’re watching someone on Zoom and you can’t quite see their legs, or the head, or the bits in between that’s a bit more difficult, but it was fine. It was fine” (P6)

## Living with chronic respiratory disease

### Having coping mechanisms

Individuals living with chronic respiratory disease had developed coping mechanisms to deal with symptoms, being able to leave their homes, or altering the way they think about their condition, by accepting their disease.

### Frustration

Living with a respiratory condition was frustrating for participants which led to very low mood and depression. Part of the frustration came from a lack of understanding of the condition, both from others or participant’s own understanding about what they have previously been informed about their disease.

### Knowing own boundaries

Participants knew their boundaries related to their physical function and associated symptoms such as breathlessness. Individuals didn’t see these as hugely limiting. Some saw these boundaries as a challenge, others perceived it as just having to get on with their illness.

### Lack of respiratory care in the NHS

Participants reported a lack of care provision from medical professionals and specific weaknesses in the NHS system by focusing on the provision of treatments in the absence of sufficient information to enable people to understand why those treatments are needed. Furthermore, participants did not understand why social groups were not promoted as much within general practice. This is particularly relevant in the context of the social prescribing agenda within the NHS Long Term Plan.^18^

## Main themes of “BreatheHappy”

### Valued social side

The social benefits of being a member of the “BreatheHappy” group were wide ranging. The basic interaction with others which was rare for those who otherwise didn’t socialise in the week was valued, and it was seen as a particular benefit to socialise with those who had a shared understanding of lived experiences of respiratory disease, which provided a basis for friendship.

People had never knowingly had such an opportunity to meet others with similar conditions before. The social element of “BreatheHappy” provided the main enjoyment from the programme.

Being with others who are similar in perceived ability was seen as confidence building, creating a safe space of belonging.

### Disease education beneficial for self-help

Educational talks and the information provision from Healthcare Professionals within “BreatheHappy” was emphasised a lot. Many participants were eager to learn about their condition and know how to help themselves and felt this was achieved in the course. Participants commented that they often forget advice, so the information that was provided at “BreatheHappy” provided a good reminder and the learning was both from HCPs and from each other.

### Improved mental Health

Participants felt like they gained a more positive outlook on life and improved mood, leading to a greater sense of well-being (mental health), but generally no change to physical health from “BreatheHappy”. The improvements in mental health were discussed in a way that was the main driver of well-being improvement compared to physical health improvements. “BreatheHappy” motivated people to do more.

### Supportive leadership

The healthcare professional leader of the group was hugely influential regarding the benefit people got from the group and also a reason to keep attending. The participants valued her approachability and the way she approached educating participants with precise explanations. The group leader had over 2 decades of experience treating people with chronic respiratory diseases and participants described her as “special” to the extent that the impact of the programme may not be the same with another leader running “BreatheHappy”.

### Logistics of BreatheHappy

A number of logistical issues were brought up by participants. These are useful considering future research design or implementation. The location of the group was difficult for some as participants deemed it far to travel (over 15 min). This was merely out of preference for the majority, an individual suggested they would prefer a location closer to their home enabling them to walk, extending the physical benefits. However, for those who relied on others to drive them, the location was a greater inconvenience. This was discussed in combination with a suggested reduced frequency of the programme but an extension of the time the groups ran to 3 h a fortnight. Other logistical considerations included the consideration of the digital provision of “BreatheHappy”. Participants were content that some digital provision was provided, but suggested that this should be as a back-up option as a face-to-face group was preferred and could follow instructions easier.

#### Practicality and Implementation

Nine participants (six females) with an average age of 72.2 years (SD: 10.6) consented to the study. Table 3 shows the participant baseline demographics.

**Table 3.** Baseline demographics.

Baseline demographic	Number of participants
Gender (Male:Female)	3:6
Age (yr)	
50-59	1
60-69	2
70-79	3
80-89	3
Ethnicity	
White British	8
White Irish	1
Respiratory condition	
COPD	1
Bronchiectasis	4
Asthma	1
Pulmonary Fibrosis	1
Bronchiectasis and Asthma	1
COPD and pulmonary Fibrosis	1
Time since diagnosis	
<5 years	2
5 - 10 years	1
11 – 20 years	1
21 – 30 years	4
31 – 40 years	0
41 – 50 years	0
51 – 60 years	1
Previous participation in PR	
Yes	3
No	6
Employment status	
Retired	8
Employed, part-time	1
Living status	
With partner	6
Alone	3
Other social group participation	
Yes	6
No	3

COPD, Chronic obstructive pulmonary disease; PR, pulmonary rehabilitation.

Participants were 22.2 years (SD: 16) post diagnosis and had a variety of obstructive and restrictive respiratory diseases. Three participants had previously participated in PR. Five participants reported no exacerbations in the previous 12 months, one participant reported one exacerbation, two reported two exacerbations, and one reported three exacerbations. Eight were retired. Six participants lived with a partner and six also participated in other social groups including singing for lung health, harmonica, University of 3^rd^ Age, zumba, tennis, tai chi, knit and natter, and Breathe Easy.

The programme was not implemented as planned. Originally the programme was planned to be delivered for 14 sessions. Only 10 sessions were delivered (see programme table in appendices). Those that were not delivered have been grey-filled on the online programme table. The start date of the programme had to be pushed back by 2 weeks as there were administrative and logistical issues with equipment purchasing from the budget, and availability of the hall. It was envisaged that we could ‘add' the sessions on at the end. However, a clinician group leader (EBS) moved jobs and we did not have the staff to continue, and so the programme was curtailed.

Attendance at the programme was good. Out of the 10 sessions provided, two participants completed 10, three completed 9, two completed 8, one completed 7, and one completed 6. One patient missed two of the sessions due to holiday, one patient missed two sessions due to illness, two patients missed a session each due to an outpatient appointment.

The majority of participants completed all outcome measurements (Tables 4 and 5). Participants were given questionnaires to complete at the end of the programme. Some were unable to complete them during the final session due to time limitations. These individuals were given the questionnaires to return by post but failed to do so. One participant completed their PA and SB monitoring a week later than others at the end of the programme. This participant was unable to attend the final session (as they were on holiday). While it may have been possible for the participant to take the monitors with them, also it was felt that this would not have been representative of the baseline data. Therefore, it was decided that having a later collection date would provide a more valid representation of the participant’s habitual activity.

**Table 4.** Group mean data for quantitative psychological, functional, and quality of life measures.

Outcome variable	Pre-intervention	Post-intervention	Mean difference (95% CI)	*P*-statistic (effect size)
Hand grip, DH (kg) [*n* = 7]	23.0 ± 7.0	24.7 ± 11.3	1.7 (−6.7 – 10.0)	0.646 (0.18)
Hand grip, NDH (kg) [*n* = 7]	20.9 ± 8.1	20.6 ± 9.3	−0.3 (−3.4 – 2.9)	0.847 (0.08)
TUG (s) [*n* = 7]	8.13 median (IQR 7.48)	9.25 median (IQR 0.72)	0 median (IQR -0.40 – 0.40)	0.866
CHI (total score) [*n* = 6]	17 ± 8	17 ± 6	0.3 (5.3 – 6.0)	0.885 (0.06)
CAT (total score) [*n* = 8]	20 ± 6	18 ± 7	−1.5 (−4.8 – 1.8)	0.324 (0.38)

IQR = Interquartile range; DH = dominant hand; NDH = non-dominant hand; TUG = timed up and go test; CHI = COPD Helplessness Index; CAT = COPD Assessment Test.

**Table 5.** Group mean data (*N* = 7) for average daily sedentary and physical activity behaviours baseline- and end-intervention.

Outcome variable	Baseline	End-intervention	Mean difference (95% CI)	*p*-value (effect size)
Sedentary Behaviour				
Total sedentary time (mins)	569 ± 96	581 ± 98	12 (34–59)	0.536 (0.248)
Step count	7523 ± 3629	8807 ± 3849*	1284 (240–2329)	0.024 (0.988)
Stepping time (mins)	96 ± 40	112 ± 45*	16 (5–27)	0.011 (1.36)
Sitting time (mins)	520 ± 94	506 ± 71	−15 (-31–60)	0.462 (0.297)
Number of sit to stands	50 ± 9	53 ± 15	3 (-9–4)	0.410 (0.335)
Sedentary bouts <30m (mins)	261 ± 27	256 ± 62	−5 (-58–68)	0.896 (0.069)
Sedentary bouts ≥30m, <1h (mins)	135 ± 27	161 ± 36*	26 (0.2–52)	0.049 (0.931)
Sedentary bouts ≥1h,<2h (mins)	105 ± 30	110 ± 64	−6 (-42–54)	0.770 (0.116)
Sedentary bouts ≥2h,<4h (mins) ǂ	33,30	0,30	−42 (-243–16)	0.075 (1.485)
Physical Activity				
Light intensity PA (mins)	155 ± 41	180 ± 44*	23 (6–38)	0.006 (1.6)
Moderate intensity PA time (mins)ǂ	68,34	72,46	10 (-15–35)	0.128 (1.739)
Vigorous intensity PA time (mins) ǂ	0.3, 3	0.3, 1	0.24 (-5 – 4)	1.000 (0.000)

* = significant difference; h = hours; m = meters; min = minutes; PA = physical activity; Group mean (±SD) data are reported as the daily average of valid days baseline- and end-intervention. Sedentary Bouts ≥2h,<4h (mins); Moderate and Vigorous Intensity PA Time (mins) are non-parametric and presented as median and IQR, with effect sizes calculated as Pearsons r denoted by^ǂ^. Intensity classifications are defined as; sedentary (<1.5 METs), light (1.5 – 3.99 METs), moderate (4.0 – 6.99 METs) and vigorous (>7 METs). Note: Effect sizes are not calculated for non-parametric data.

##### Programme uptake, costs, and adverse events

The programme was accessed via self-referral and was advertised via social media in a small rural town where the location was based. In the next small rural town was a local Asthma and Lung UK Breathe Easy group and members were invited via leaflet distribution. The local surgery and social prescriber were informed of the study, sent a poster and invited to signpost patients. Recruits came from social media and Breathe Easy, but the local surgery did not engage with the invite to signpost patients.

No adverse events or unforeseen issues occurred. The outcome measures proved acceptable to patients with no concerns raised. The activity monitors were acceptable and patients found them simple to use.

The resources required for the programme itself were room hire costs for the sessions (£15 per hour x 28 = £390), plus staff costs equivalent to Agenda For Change x1 band seven and x1 band 6 - 30 h each, total staff approx. £1700, plus £50 for stationary and refreshments. Therefore, total costs were approximately £2130 per programme.

The programme demonstrated good practicality but was not implemented as planned due to limited resources and logistical and administrative issues.

#### Limited efficacy testing

As shown in Table 4, there was no statistically significant change in measures of strength, functional mobility, psychological health status, and disease burden. Two patients did not attend the final session. We were unable to collect the physical function tests for these patients due to the finite number of sessions and the questionnaires sent for self-completion were not fully completed.

#### Physical activity and sedentary behaviour

Table 5 presents physical activity and sedentary behaviour data pre- and post-intervention (*n* = 7; >5-valid days included in analysis for all participants – all data available in Online Appendix). Following 10 weeks of the “BreatheHappy” intervention, there was a significant increase in daily step counts, stepping time and the number of minutes completing light physical activity. There was also a statistically significant increase in sedentary time between 30 min and 60 min. There was no significant effect of the intervention on any of the other physical activity (moderate, vigorous activity) or sedentary behaviour (standing time, total sedentary time, sitting time, sit to stands, sedentary time 60-120 min) outcome variables. However, Sedentary Bouts ≥2 h,<4 h were reduced. This indicates a potential change in activity pattern of participants as a result of the “BreatheHappy” intervention.

## Discussion

The overall aim of this study was to pilot the model of “BreatheHappy” to determine acceptability, practicality, implementation, and preliminary markers of efficacy to highlight areas of the methodology which need improving or changing in preparation for a future definitive study (adaption).

### Acceptability

“BreatheHappy” was acceptable according to the participant interview data. “BreatheHappy” offers a space where people with shared experiences can learn from each other and be educated in their condition, similar to PR. This group also valued the disease education component of “BreatheHappy” and the majority hadn’t previously participated in PR.

### Practicality and Implementation

The programme was largely practical regarding participant attendance and completion of outcome measures, but neither were 100% due to issues perhaps common in chronic disease research. The programme was also practical from a safety perspective as no adverse events were reported. However, the “BreatheHappy” programme was not implemented as planned as the programme was curtailed to 10 sessions rather than the 14 planned.

### Efficacy testing

Participants improved step count beyond the minimal clinically important difference available for people with COPD.^40^ Step count volume and pattern is also similar between those living with COPD and ILD.^41^ The step count change is beyond the daily minimal difference shown in other studies,^42^ and so there is likely a clinically relevant step count change. Furthermore, in our study we observed a mean increase in step count of 1284 steps/day, whereby an increase of 1000 steps per day is clinically meaningful in other studies.^43,44^ The primary outcome for a future randomised controlled trial will be based on the physical activity and sedentary behaviour outcomes, likely step counts or minutes in light intensity physical activity given the clinical significance, and are outcomes directly linked to the aims of the intervention. However, our data also show an increase in shorter bouts of sedentary behaviour. This may indicate that individuals are pacing their activity better over a daily period, by both increasing activity in bouts interspersed with increased short rest periods, with reduced longer rest periods.

Although there was a significant improvement in physical activity outcomes, it did not match a similar magnitude in other outcome measures, such as TUG and handgrip strength. Previous research has reported that there is not a strong correlation with physical activity and other health related quality of life, physical performance or capacity outcome measures in chronic respiratory disease.^41,42^

Our research indicates that the main mechanisms of effect on physical activity levels may come from the combined social and mental health benefits gained from the programme, as made clear from the qualitative data. There is likely a bidirectionality of effect between physical activity and social activity. Those who do physical activity exhibit more prosocial behaviours.^45^ Furthermore, evidence suggests that social interactions between low to moderately active individuals are the most important social factor that influences low active populations to become and stay physically active.^46^ In our study here, those individuals who were already socially active were showing reasonable baseline levels of physical activity, and may have been primed for the potential benefits of further social activity and therefore further gains in physical activity. Social networks are positively associated with steps per day, with those with fewer social ties walking 1500 steps per day less than those with greater ties,^47^ which adds weight to this theory. In this regard “BreatheHappy” could be recommended for people who complete PR. There is also likely to be a compounding beneficial effect when improvements in mental health are perceived, as light intensity physical activity is inversely associated with depression.^48^

### Adaptions

The qualitative findings suggest keeping a focus on the social nature of the activity choices in the programme because the social side was so valuable. As participants learned from each other, the format of the education provided in future trials should include group-based discussion, encouraging people to discuss their lived experiences of chronic respiratory disease around different topics, which could be facilitated appropriately by an HCP. Considering the social and mental health benefits reported in the qualitative data, associated outcome measures should be used in future studies. It is interesting that an improvement in function or symptoms was not a theme generated from participant data. This matches the lack of significant improvement in associated quantitative outcomes. This may also demonstrate the robust qualitative data analysis method which was inductively focused and not driven by what the researchers’ potential biases were about the likely impact on lived experiences.

It is likely that multiple venues in areas with good transport links, or providing specific funding for patient transport will be needed in a future study. Clinicians with vast experience of running group-based rehabilitation need to be recruited to run the “BreatheHappy” groups in future studies and should also be costed appropriately.

In future studies we aim to recruit those who may also not already be very socially active. In this population the need for the intervention, and the gains in outcomes achieved may be greater. Individuals who are socially disengaged are more likely to have worse health outcomes and be more functionally disabled.^49^ In context of participants already being socially active, our findings highlight the focus in “BreatheHappy” on the integration of physical activity within social activities that were made available on the programme. To more definitively determine the effect of the physically active nature of the social activities in “BreatheHappy”, a future randomised controlled trial could provide a control group that offers social activity in the absence of physical activity, such as a film club.^50^ Like other research published in the field, the social benefits of being with others with respiratory disease is clear.^51–53^

The other quantitative outcomes chosen for a future study could be further revised, potentially including measures of social isolation. Field-based walk tests are likely more relevant for lower limb endurance and functional capacity compared to the functional outcome measure of hand grip strength, which was thought potentially viable in context with the measures relationship with mobility related quality of life^54,55^ but we acknowledge its potential lack of responsiveness.

There are some logistical issues to consider regarding future trial design. These include the format (face-to-face vs online delivery), location and frequency of the classes. Other potential viable locations include non-medical community spaces, in particular a local church hall which has suitable social facilities. Furthermore, multiple recruitment channels including social media, local BreatheEasy groups, and directly from local GP practices may be required in future studies. Strategies on how to better engage local GP practices in promoting such groups is needed. Recruitment could also be more focused on people who have not previously participated or declined pulmonary rehabilitation, and for those who have completed PR who have developed a social network and become accustomed to the potential benefits of physical activity. This may maximise the likely benefits from attending “BreatheHappy”. Furthermore, different frequencies of delivery need to be trialled to both determine dose response such as studies performed in pulmonary rehabilitation,^56^ but also balanced with suitable patient adherence. A twice weekly programme is likely to be more effective, but perhaps less attractive to those more socially active. Funding for research staff to support clinicians in patient data collection will ensure more robust completion of outcome measures.

## Strengths

The strengths of the programme are its community setting and minimal equipment training, which are more likely to increase accessibility to rehabilitation and translate into independent maintenance of physical activity.

The differentiation in this study, compared to many of those in PR, is the ethos that increasing one’s physical activity is beneficial in the absence of exercise prescription, like in PR. The word “training” may imply the basis of exercise prescription principles being delivered. We used training here in a sense of developing skill and confidence in activities which relate to the competency of physical activity maintenance as a long-term behaviour outcome. To support this, the programme provided education on participating in physical activity by an HCP. In this way it is somewhat aligned to the purpose of PR regarding long term health promoting behaviour change. The intervention could be interpreted as a low-resource exercise training. For this to be the case the social physical activities need to be individually prescribed based on an assessment that is fit for purpose for the range of social activities available.

## Limitations

This study design did not include a control group or sham intervention. Therefore, the effectiveness of the intervention cannot be shown at this stage. The step count increase may have been a result of simply wearing the accelerometer.

The sample size is small and likely has a self-selection bias, therefore limiting generalizability. Due to the funding limit received, this pilot work could not be extended in time to logistically run the group with a second block of participants, nor resource for further quantitative or qualitative data collection. All statistical analysis therefore lacks power to determine significant differences. However, the collection of quantitative data in the pilot stage also enabled other feasibility outcomes to be assessed (i.e. completion in setting). The CAT score had a 1.5 point difference which is similar to the difference shown previously to be statistically significant in group-based singing for lung health intervention with a larger sample.^57^ This does not meet the recognised MCID for PR, but an MCID is specific to the intervention delivered. Further work is needed to establish an MCID for outcome measures from this “BreatheHappy” intervention.

Most individuals in this study were already socially and physically active and so the intervention was perhaps delivered to those who would not benefit the most from the physical impacts and social connectivity.^49^

Staff were not interviewed about their experiences of running the programme in order to determine the feasibility of “BreatheHappy” delivery and outcomes from their perspective. Further studies should consider collecting data about staff experiences and a health economic analysis.

The intervention delivered here did not provide a home-based programme, which could offer more accessibility and flexibility for participants. Having the choice to participate in all formats of the programme would be ideal, if and once all are made available. Participants were given access to a digital self-management intervention to compliment face-to-face provision but we did not offer a digital intervention option as a sole intervention choice here. We have piloted a digital version of rehabilitation which could be transferrable to the “BreatheHappy” model for a telerehabilitation adaption for future study.^58^ However, it is likely that the social nature of the intervention would be lost if activities were delivered one-to-one in patient’s homes.

The “BreatheHappy” intervention, if proven clinically effective in future studies, could be considered as a choice in a range of evidence-based group activities. PR is the gold-standard intervention recommended for patients with chronic respiratory disease and should participants be eligible then we would still recommend referral to a local PR programme.

## Conclusion

This study investigated the acceptability, practicality, implementation and efficacy testing of a “BreatheHappy” group-based social and physical activity intervention to determine feasibility. The programme showed aspects of feasibility worth taking forward because it was acceptable from the participants’ experience, practical because of the participation rates and the majority completed the battery of outcome measures. There were signs of efficacy in step count, light physical activity improvement and change in sedentary behaviour pattern. However, the programme was not implemented as planned and adaptions will need to be made for a future study.

## Supplemental Material

Supplemental Material - The acceptability, practicality, implementation and efficacy of a physical and social activity intervention ‘BreatheHappy’ for people with long-term respiratory conditions: A feasibility study Supplemental Material for The acceptability, practicality, implementation and efficacy of a physical and social activity intervention ‘BreatheHappy’ for people with long-term respiratory conditions: A feasibility study by A Lewis, LA Turner, S Fryer, R Smith, H Dillarstone, YW Patric and E Bevan-Smith in Chronic Respiratory Disease

Supplemental Material - The acceptability, practicality, implementation and efficacy of a physical and social activity intervention ‘BreatheHappy’ for people with long-term respiratory conditions: A feasibility study Supplemental Material for The acceptability, practicality, implementation and efficacy of a physical and social activity intervention ‘BreatheHappy’ for people with long-term respiratory conditions: A feasibility study by A Lewis, LA Turner, S Fryer, R Smith, H Dillarstone, YW Patric and E Bevan-Smith in Chronic Respiratory Disease

## Data Availability

Data can be made available on reasonable request.
