# Supplementary material for: The acceptability, practicality, implementation and efficacy of a physical and social activity intervention ‘BreatheHappy’ for people with long-term respiratory conditions: A feasibility study
Source: Chron Respir Dis. 2024 Mar 29;21:14799731241238435. doi: 10.1177/14799731241238435 (PMC10981237; doi:10.1177/14799731241238435)
Supplement: Supplemental Material - The acceptability, practicality, implementation and efficacy of a physical and social activity intervention ‘BreatheHappy’ for people with long-term respiratory conditions: A feasibility study [file sj-pdf-1-crd-10.1177_14799731241238435.pdf]

Appendices

Semi-structured Interview Guide

Welcome

- Welcome/name introduction/thanks for participating
- Run through participant information sheet and consent form
- Please feel free to pause for a drink or if you need to take a break at any point that's fine, if there are any questions you don't feel comfortable about answering just let us know and we can move on
- *begin recording* explain you will be recording

Warm-Up Question (gaining rapport with interviewee):

- How are you feeling today? (i.e., if it's hot – ask how they finding the heat)

Introduction:

I am aware you have taken part in the BreatheHappy programme. This discussion is part of the research the university is doing to look at whether BreatheHappy is helpful to people with respiratory conditions. We have no affiliation to BreatheHappy so please feel open to discuss your true thoughts/feelings without fear of judgement, there is no right or wrong answer.

The questions are designed to give you the space to open up and direct your answers in whichever way you interpret the question. We are ultimately interested in you and getting to know about your experiences so we hope that you will feel open to speak at length. You are able to take your time and think about your answers, there might be some moments of silence throughout the interview where we give you time for this and we will prompt you occasionally when necessary, but we hope that the conversation will mostly be guided by your answers. Also, I will likely take a few notes throughout so please don't be alarmed if I'm tapping away or writing something down, I am still listening!

Do you have any questions before we begin?

Opening Question:

- Would you like to tell me about your experience of your lung condition?
  - Prompts:
  - What does it feel like having your lung condition?
  - How do you look at the world around you when you have "lung condition"?

- What does a good day look like for you?
- What does a bad day look like for you?
- Tell me about how you experience your lungs in relationship with your general health (physical, mental, social)
- How do you cope with your lung condition?
- Reflect on their past and future selves
- How do you approach being with others with your lung condition?
- Can you tell me more about your experience of the Monday sessions?
- How did you feel when you first started Breathehappy and has this changed since?
- What would you tell others with lung conditions about BreatheHappy?

- Focused questions (*only after giving them space to talk about everything that's important to them*, summarise their themes back to them e.g. you mentioned * and get them to confirm):
  - That's really interesting, can you tell me more about that?
  - What do you mean by ?
  - How does that make you feel?

Participant Reflection on BreatheHappy:

- Why do you attend BreatheHappy?
  - Prompts (if struggling): How does it make you feel?
- Could you describe a meaningful experience from BreatheHappy that comes to mind?
  - Prompts: Why was this experience important to you? Can you describe any other interesting experiences?
- Have you noticed any personal changes since joining BreatheHappy ?
  - Prompts (if struggling): body perceptions and their relationship with their lungs, mental, emotional, social, spiritual, interactions with environment, time-oriented. Interactions with staff and other group members.
- How do you experience movement and physical activity now?
  - Prompt: what happens with your breathing during (activity)? How do these experiences compare to before you joined Breathehappy?
- Is there anything you would change about BreatheHappy?
  - Prompt if participant attends other groups: In consideration of other groups you attend?
  - Prompts (if struggling): Is there anything you find challenging?
  - E.g. Timings/length of sessions? Number of people attending? Location? Exercises?
- When it was hot and the session was online how did you find that session?*

Concluding Questions

- Do you see yourself continuing to attend BreatheHappy?
  - Prompts: Why/why not?
- Is there anything that we haven't covered during this interview that you'd like to share about your BreatheHappy experience or living with a lung condition?

Thank you for your time today.

General prompts:

- Can you tell me more about
- What do you mean by
- How does that make you feel?
- Why?
- When?
- Who?
- How?

Programme Development

Previously, a community "Breathehappy" walking and self-management group ran by one of the authors (with experience of running PR groups) under the National 'walking for health' scheme was noted to have many life changing impacts on people who had both previously declined / not completed or who had completed PR. The self-management elements developed through co-creation of empowering healthy lifestyle activities, which ran in tandem with the walking group such as navigating different outdoor terrains, dealing with breathlessness in outdoor spaces and using Tai Chi to elicit increased confidence and balance on walking.

As part of an ongoing co-creation process, participants, through group discussion, reflected on what could be included in the group sessions to help their wellbeing. The process of co-creation in itself was felt to be therapeutic as participants felt that they were contributing to and helping to design / create and intervention to help themselves. This increased their feeling of self-worth. The participants reported that they liked the fact that the group was not medically focused and that they were not being 'prescribed exercise'. They were undertaking a pleasurable activity with others who faced similar difficulties but they felt safe as a Healthcare Professional present. Self-management activities were based on the education component of a PR programme, but delivered in a far more creative and person-centred way than in traditional PR. For example, gamification of complex physiology in order to simplify concepts. The health education topics were more holistic than in a traditional PR programme, encompassing aspects such as feelings experienced at diagnosis and disease adaptation. Patients welcomed the approach of being accepted in the group 'just as they were' without expectations of physiological improvement. They felt this reduced the 'pressure to improve' as exists in PR, which focusses heavily on the evidence based physiological outcome of exercise capacity. Breathehappy was somewhere where they could 'simply be' and learn how to still enjoy life despite their lung condition. Participants described this collectively during group

conversations as they cornerstone of the group. Participants reported increased confidence, feelings of self-worth and belonging. They spoke of no longer being ‘defined by their health condition’ and a reduction in the feeling of vulnerability that they reported as being a major contributing factor to a reduction in physical and social activity. These outcomes were translated into increases in not only the distance participants were able to walk, but also that they had the confidence to go walking in places they previously were nervous about walking to. Interestingly the group inspired a small number who had previously declined PR to undertake a programme.

The “BreatheHappy” walking / social group regrettably was not undertaken as a research study, however such observed excellent outcomes from this intervention warranted further exploration to see if it could be replicated in a different community location. A very small research grant was obtained from a local university. The grant enabled a university-led programme which was delivered by 3 members of university staff from health and exercise science: a registered RNS with extensive previous PR experience, a sports science specialist well-published in measuring PA in sport and health, and a registered paramedic. The sessions had 2 members of staff present. The previous “BreatheHappy” location was in a small community hall adjacent to a park which enabled enjoyable outdoor walking along with a dry space for other indoor activities. Learning from this group enabled construction of a delivery model which potentially could be more easily described / defined. The elements were included in the more structured model which had been deemed by patients to have the most powerful impact on their lives. Part of the previous success of “BreatheHappy” was the informality of the sessions which were often patient-led with the HCP as a facilitator rather than director. Thus, the structure was given adaptability to be flexible. Much debate occurred during programme development about appropriate outcome measures and whether any measures of exercise tolerance should be included given that we were not directly comparing “BreatheHappy” to PR.

Detailed “BreatheHappy” Programme Structure

Each session comprised of a ‘meet and greet’ including brief informal chat about progress, a section of varied physical activities, followed by social time with refreshments and finishing with a group leader-led health educational activity. Physical activity mainly comprised of walking around on of three routes in the outside space of the community hall. This was prescribed based on the patient’s TUG test result combined with a subjective assessment of their efficacy for walking outdoors. Participants were encouraged to embrace what they had learnt in TMW in identifying their own ‘soft limits’ in order to set their own walking pace. A soft limit in TMW is where the person is operating at a level where they are balanced and grounded but still working at their full potential. Walking sports such as rounders and croquet were introduced to enable participants to engage in activities that elicited movement and breathlessness but remained fun. During social time participants had refreshments together and engaged in open conversation. Each session was delivered by a respiratory-trained healthcare professional. The approach of the programme was based on an ethos of peer support, mindfulness, socialisation, enjoyment, developing confidence and decreasing the feeling of vulnerability. Patients were taught the benefits of activity and encouraged to walk and move about more in-between sessions.

The first hour of each session was devoted to a menu-based physical activity session, one of which was usually walking outdoors and the second hour devoted to group activities that either focus around activities designed to help participants select healthy behaviours, or discussion groups that elicited positive adaptation to ill health through activities such as group problem solving.

Physical activity

A flexible menu of indoor and outdoor physical activities was available ranging from low to moderate intensity. The activities offered each week was dependent on the abilities and desires of the attendees. Two activities per session were available and participants chose, with support from their rehabilitation plan, which activity they participate in. Activities included:

Tai Chi movements for wellbeing (indoor, sitting or standing). (an evidence based deconstructed adaptation of Tai Chi suitable for all levels of disability).

Outdoor or indoor team 'walking' games: for example, soft rounders, soft cricket, frisbee, parachute games.

Indoor moderate intensity aerobic exercise.

Indoor low intensity resistance exercise

Indoor or outdoor low intensity activity requiring gentle movement: for example the activities below which require movement.

TMW

TMW or 'Tai Chi movements for wellbeing' is a sequence of movements informed by the principles of Tai Chi and Chi Kung without the complexity of the traditional forms. The sequence has been specifically designed to promote physical and emotional health and wellbeing. The sequence of movements is suitable and safe for people with COPD and can be done standing or sitting. TMW is based on some core understandings. Firstly that there is a relationship between the body and mind and the way we move physically expresses our emotions. Secondly the notion of a 'soft limit' where we have confidence to mildly challenge ourselves without going outside our comfort zone, and thirdly 'mirroring' where strength is reigned in to match vulnerability, rather than vulnerability

causing an imbalance. These understandings are expressed through the movement sequence. TMW will be used as an adjunct to walking, the trainer applying the core understandings and principles to the way the participants work to reduce their vulnerability walking outdoors. It is hypothesised that the application of TMW to walking will help participants build confidence, increase stability and walk to an intensity that is within their 'soft limits'. An example of how this will be applied is 'mirroring'. Rather than slower walkers feeling disillusioned by having to keep up with faster walkers one of the walk leaders will walk at the same slow pace, 'mirroring' the participant. It is envisaged that this will build self -esteem and a give the participant a sense of achievement rather than failure. TMW will incorporate therapy for breathelsness, anxiety and panic, poor self-esteem and negative thought patterns. During the first six weeks of the "BreatheHappy" programme participants will have group instruction in TMW. During weeks 6 – 12 participants will perform the TMW sequence as a group. Participants will be invited to practice TMW regularly throughout the week in-between the "BreatheHappy" sessions.

Outdoor walking

The venue provided opportunities for outdoor walking that increase incrementally in their level of challenge. Part of the challenge of outdoor walking is the difficulty in the different terrains, reading maps and negotiating obstacles such as stiles or mud. Chronic health conditions increase the feeling of vulnerability which may magnify these challenges. An assessment of the walking route distances combined with the difficulty of the terrain enabled routes to be graded incrementally. 'BreatheHappy' participants helped the researchers to determine grades by walking the routes. This enabled grading from the perspective of a person living with a lung health condition so that the grading was a truer reflection of increment level specific to people with chronic respiratory disease. Following the initial health assessment participants were matched by ability to a graded outdoor walking route. If they were not able to walk safely outdoors then they were assigned to the lower

intensity activity indoors. Throughout the programme, if they increased in confidence and ability, they were gently invited to move to the next incremental walking route. They were encouraged to enjoy their environment and the company of others who may walk with them. The timing for the walking routes ranged from 15 minutes to a maximum of 1 hour to allow for diversity of ability and walking route.

During inclement weather participants were still invited to walk outdoors tailoring their route, timings and clothing accordingly. In between sessions, participants were invited to try to increase the frequency of outdoor walking rather than speed or distance.

Group activities

Group activities were designed to elicit motivation and activation within participants in order to select healthier physical, psychological and social behaviour. Traditional educational didactic talks about health topics were replaced with group participatory learning activities which were designed to be fun, interesting and engaging for participants. Example activities are as follows:

- Group problem solving
- Discussion / debate
- Cookery sessions
- Craft sessions
- Quizzes
- Mindfulness exercises
- Drawing / painting
- Games
- Photography
- Gardening

Using these example activities as a delivery vehicle, the educational element focuses on living well with illness through healthy personal adaptation strategies and developing positive health behaviours.

Using these example activities as a delivery vehicle, the educational element focuses on living well with illness through healthy personal adaptation strategies and developing positive health behaviors. BreatheHappy utilises a unique conceptual model ‘COPE’ that facilitates participant understanding of the principles of healthy adaptation to chronic disease. ‘COPE’ is an acronym as follows:

Control: **O**ptimise: **P**revent: **E**ngage and topics are organized into those 4 principles. The topics list below is an example:

**Control**<br><br>Moving the locus of health control to the participant	Pathophysiology of Respiratory Diseases<br><br>Mindfulness<br><br>Anxiety and panic management<br><br>Pelvic floor exercises<br><br>Navigating the health and social care system
**Optimise**	Sputum management<br><br>Dealing with exacerbation

Ensuring that treatment is optimized and physiological 'environment' is in optimum condition to allow treatments to work.	Medication<br><br>Risk reduction
**Prevent**<br><br>Evidence based strategies to prevent exacerbation of health condition	Recognition of exacerbation<br><br>Risk factors for exacerbation
**Engage**<br><br>Reducing the impact of illness on connection with society	Dealing with altered body image Breathlessness management<br><br>Energy Conservation<br><br>Financial help<br><br>Safety when away from home

Group Therapy

The social solidarity and therapeutic relationship between participants and healthcare professionals was utilised to its full potential. Staff members showed and elicited an environment of care and compassion for all members, in order that participants experience a sense of belonging and purpose.

Timetabled all sessions

Session 1<br><br>***30/5/22***<br><br>***13.30 – 15.30***	***15 MINS***<br>*Meet and greet*<br>*Icebreakers*<br>*Groundrules*<br>*Session briefing*				

Session 2<br>6/6/22	***15 MINS***<br>*Meet and greet*<br>*Icebreakers*<br>*Groundrule s*<br>*Session briefing*	***15 MINS***<br>*TMW & SOB management*	***30 MINS***<br>***ACTIVITY 1:***<br>*Outdoor walking*<br>***ACTIVITY 2:***<br>*Indoor games*	*Refreshment s / peer support*	***45 mins: Group therapy / education***<br>*Introduction to BreatheHappy*
Session 3<br>13/6/22	***15 MINS***<br>*Meet and greet*<br>*Session briefing*	***15 MINS***<br>*TMW & SOB management*	***30 MINS***<br>***ACTIVITY 1:***<br>*Indoor moderate intensity exercise*<br>***ACTIVITY 2:***<br>*Cookery*	*Refreshment s / peer support*	***45 mins: Group therapy / education***<br>*Reducing vulnerability during physical activity*
Session 4<br>20/6/22	***15 MINS***<br>*Meet and greet*<br>*Session briefing*	***15 MINS***<br>*TMW & SOB management*	***30 MINS***<br>***ACTIVITY 1:***<br>*Outdoor walking*<br>***ACTIVITY 2:***<br>*Container gardening*	*Refreshment s / peer support*	***45 mins: Group therapy / education***<br>*Nutrition and respiratory disease*
Session 5<br>27/6/22	***15 MINS***<br>*Meet and greet*<br>*Session briefing*	***15 MINS***<br>*TMW & Anxiety and panic management*	***30 MINS***<br>***ACTIVITY 1:***<br>*Outdoor walking*<br>***ACTIVITY 2:***<br>*Indoor games*	*Refreshment s / peer support*	***45 mins: Group therapy / education***<br>*Pelvic floor exercises*
Session 6<br>4/7/22	***15 MINS***<br>*Meet and greet*<br>*Session briefing*	***15 MINS***<br>*TMW & Anxiety and panic management ent*	***30 MINS***<br>***ACTIVITY 1:***<br>*Indoor moderate intensity exercise*<br>***ACTIVITY 2:***<br>*Craft activity*	*Refreshment s / peer support*	***45 mins: Group therapy / education***<br>*Navigating the health and social care system*
Session 7<br>11/7/22	***15 MINS***<br>*Meet and greet*<br>*Session briefing*	***15 MINS***<br>*TMW & Anxiety and panic management*	***30 MINS***<br>***ACTIVITY 1:***<br>*Outdoor walking*<br>***ACTIVITY 2:***<br>*Movement for relaxation*	*Refreshment s / peer support*	***45 mins: Group therapy / education***<br>*Sputum clearance*
Session 8<br>18/7/22	***15 MINS***<br>*Meet and greet*<br>*Session briefing*	***15 MINS***<br>*TMW &positive thinking*	***30 MINS***<br>***ACTIVITY 1:***<br>*Outdoor walking*<br>***ACTIVITY 2:***<br>*Indoor games*	*Refreshment s / peer support*	***45 mins: Group therapy / education***<br>*Dealing with exacerbation*

Session 9<br>25/7/22<br>? all day	***15 MINS***<br>*Meet and greet Session briefing*	***15 MINS***<br>*TMW & positive thinking*	***30 MINS***<br>***ACTIVITY 1:*** *Outdoor walking*<br>***ACTIVITY 2:*** *Movement to music*	*Refreshments / peer support*	***45 mins: Group therapy / education***<br>*Medication*
Session 10<br>1/8/22	***15 MINS***<br>*Meet and greet Session briefing*	***15 MINS***<br>*TMW & body image*	***30 MINS***<br>***ACTIVITY 1:*** *Indoor moderate intensity exercise*<br>***ACTIVITY 2:*** *Photography*<br><br>*Cookery*	*Refreshments / peer support*	***45 mins: Group therapy / education***<br>*Reducing risk factors for exacerbation*
Session 11	***15 MINS***<br>*Meet and greet Session briefing*	***15 MINS***<br>*TMW & body image*	***30 MINS***<br>***ACTIVITY 1:*** *Outdoor walking*<br>***ACTIVITY 2:*** *Indoor walking games*	*Refreshments / peer support*	***45 mins: Group therapy / education***<br>*Safety when away from home*
Session 12	***15 MINS***<br>*Meet and greet Session briefing*	***15 MINS***<br>*TMW & identifying soft limits*	***30 MINS***<br>***ACTIVITY 1:*** *Outdoor walking*<br>***ACTIVITY 2:*** *Charades*	*Refreshments / peer support*	***45 mins: Group therapy / education***<br>*Benefits you can claim*
Session 13	***15 MINS***<br>*Meet and greet Session briefing*	***15 MINS***<br>*TMW & identifying soft limits*	***30 MINS***<br>***ACTIVITY 1:*** *Outdoor walking*<br>***ACTIVITY 2:*** *Indoor moderate intensity exercise*	*Refreshments / peer support*	***45 mins: Group therapy / education***<br>*Beyond BreatheHappy*
Session 14	***ASSESSMENT***				

Schematic of sessions delivered and assessments

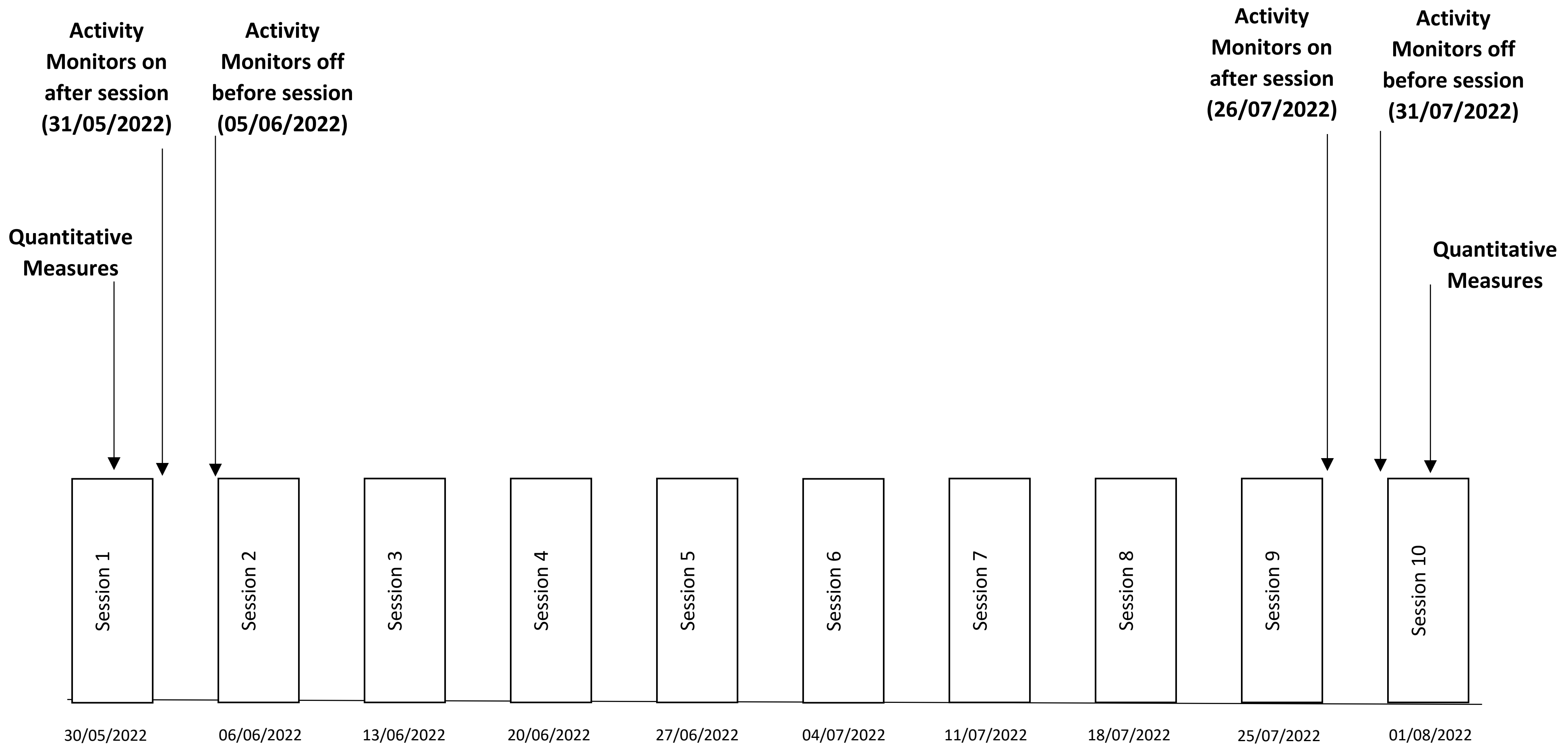

Instructions for Timed Up and Go (TUG) and Hand Grip Strength (HGS):

TUG:

Participant Instructions

When I say "Go," I want you to:
1. Stand up from the chair.
2. Walk to the line on the floor at your normal pace.
3. Turn.
4. Walk back to the chair at your normal pace.
5. Sit down again.

Researcher Instructions

1. On the word "Go," begin timing.
2. Stop timing after patient sits back down.
3. Record time

HGS:

Instructions

- Perform seated in a straight back chair with feet flat on the floor
- Assess on the participants dominant side
- Elbow should be flexed at 90°
- Forearm and wrist in a neutral position
- Set handle space for each participant
- Participant to perform 3 maximal exertions for 5s with 15 s rest between trials
- Standardised instruction to participant - "Are you ready? Squeeze as hard as you can. Harder … harder … harder … harder … harder … . relax."
